# The relationship between hydroxychloroquine plasma concentration and COVID-19 outcomes in rheumatoid arthritis patients in Saudi Arabia

**DOI:** 10.1016/j.jsps.2022.05.006

**Published:** 2022-05-23

**Authors:** Ismail A. Walbi, Hussain Alqhtani, Faleh Alqahtani, Saad Ahmed Alkahtani, Ali Mohamed Alshabi, Amer S. Alali, Hassan A. Albarqi

**Affiliations:** a Department of Clinical Pharmacy, College of Pharmacy, Najran University, Najran, Saudi Arabia; b Department of Pharmacology, College of Pharmacy, King Saud University, Riyadh, Saudi Arabia; c Department of Pharmaceutics, College of Pharmacy, Prince Sattam Bin Abdulaziz University, Al-kharj, Saudi Arabia; d Department of Pharmaceutics, College of Pharmacy, Najran University, Najran, Saudi Arabia

**Keywords:** Hydroxychloroquine, COVID-19 incidence, COVID-19 complications, Rheumatoid arthritis

## Abstract

**Background:**

The drug hydroxychloroquine (HCQ) is widely used to treat rheumatoid arthritis (RA) and has been repurposed for the treatment of COVID-19. This study aims to determine whether HCQ concentration levels in individuals with RA alter the incidence of COVID-19 or its complications.

**Methods:**

We collected plasma samples from 13 individuals with confirmed rheumatoid arthritis (RA) to measure HCQ concentration levels. The study included individuals at least 18 years old who had been taking HCQ for at least six months at daily doses ranging from 200 to 400 mg.

**Results:**

The study enrolled a total of 13 RA patients. All patients were chronic HCQ users. Among the 13 patients, 7 patients were receiving HCQ at a dose of 200 mg per day, and 6 patients were receiving HCQ at a dose of 400 mg per day. COVID-19 confirmed cases accounted for approximately 46% of all patients. Half of the infected patients (n = 3) were taking a daily dose of 200 mg daily, while the other half were taking 400 mg daily. COVID-19 symptoms ranged from mild to moderate, and the intensity of the symptoms was not severe enough to necessitate hospitalization. COVID-19 symptoms in RA patients included headache, fever, fatigue, dry cough, and loss of taste or smell.

**Conclusions:**

Our findings indicated that there was no correlation between HCQ concentrations in rheumatoid arthritis patients and the occurrence of COVID-19 or its complications.

## 1 Introduction

In December 2019, the first report of people exposed to severe acute respiratory syndrome coronavirus 2 (SARS-CoV-2) was in Wuhan, China, and it has since been reported in almost all countries (Huang et al., 2020). The WHO Coronavirus (COVID-19) Dashboard recorded approximately 400 million confirmed cases and over 5 million deaths worldwide at the time of publication (int, 2020). The emergence of SARS-CoV-2 revealed the vital need to suppress the outbreak and the critical need for an effective treatment to prevent the health system from collapsing at the onset of the coronavirus outbreak. Hydroxychloroquine (HCQ) is an antimalarial medication that has antiviral and immunomodulatory effects, making it a potentially effective treatment for SARS-CoV-2 infections. In 2003, HCQ was investigated as a potential agent for SARS, but the disease was contained before a thorough test could be conducted (Gasmi et al., 2021, Kumar et al., 2021). The observed activity of HCQ against SARS-CoV-2 in *in vitro* and clinical studies led to the rapid and widespread use of HCQ worldwide (Gautret et al., 2020a, Gautret et al., 2020b, Lagier et al., 2020, Liu et al., 2020, Pastick et al., 2020, Rosenberg et al., 2020, Yao et al., 2020, Rentsch et al., 2021).

Hydroxychloroquine is widely used in the treatment of rheumatoid arthritis (RA) (Rainsford et al., 2015). RA is a chronic autoimmune disease that affects mostly women and can affect different body joints, causing pain and loss of function. HCQ has been shown to improve survival rates, reduce the frequency of RA flares as well as organ damage, prolong disease onset, and reduce the risk of complications (Wu et al., 2018, Shi et al., 2019, Weyand and Goronzy, 2021). HCQ is administered orally in a sulfate form and is absorbed from the upper intestinal tract with 0.7 to 0.8 overall bioavailability (Tett et al., 1989). It has a long half-life, typically greater than 30 days. This long half-life has a direct effect on the drug accumulation effect and steady-state concentration, which occurs when the rate of elimination equals the rate of drug intake. According to bioavailability studies, a steady-state concentration of HCQ can be reached after approximately six months (Furst, 1996, Rainsford et al., 2015). Few studies have been conducted to determine the efficacy of preexposure HCQ in preventing SARS-CoV-2 infection or mitigating its severity. A population-based cohort study conducted in England, UK, demonstrated no substantial advantage in avoiding SARS­CoV­2 infections when HCQ was used as preexposure prophylaxis prior to the COVID-19 epidemic (Shehab et al., 2020). Other trials involving pre- or postexposure HCQ administration failed to demonstrate a reduction in SARS-COV-2 virus infection (Grau-Pujol et al., 2021, Jung et al., 2021, Rajasingham et al., 2021).

The aim of the current study was to analyze the correlation between HCQ plasma concentrations in RA patients and the occurrence of COVID-19 disease or its complications using LC/MS/MS. We believe that examining the HCQ concentration in plasma and its relation to COVID-19 incidence or outcomes will aid in our understanding of the disease's effect.

## 2 Methods

### 2.1 Materials and instruments

**Fig. 1:**
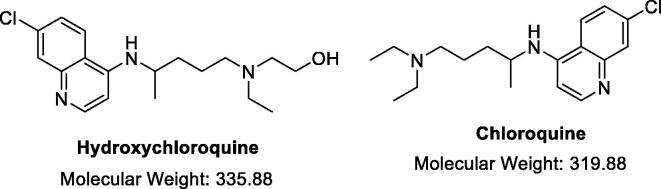
Chemical structure for the hydroxychloroquine and chloroquine (IS).

The solvents used in the current study were all HPLC grade. The reference powders and other chemicals used were of analytical grade (AR). Hydroxychloroquine sulfate (HCQ) (CAS H916900) and chloroquine diphosphate salt (CLQ) (CAS C379965) were purchased from Toronto Research Chemicals (Toronto, ON, Canada) (Fig. 1). The acetonitrile (ACN) and formic acid (FA) used in the experiment were supplied by Sigma–Aldrich Company (PA, USA). Millipore Milli-Q Plus purification equipment (Millipore, MA, USA) was employed to purify water (HPLC grade). MassLynx 4.1 software was used to control the system. Application manager QuantLynx supplied with MassLynx 4.1 Software (Version 4.1, SCN 805) was utilized to acquire, process, and report data. IntelliStart® assisted in the mass tuning process. Additionally, a rotary pump (Sogevac, SV40B1) was used to assist the vacuum, and a nitrogen generator (Peak Scientific, Scotland) was used to provide the desolvation gas. The purity of argon gas (Ar) was 99.999% and was provided by a local supplier.

### 2.2 Patient selection

We collected plasma samples from 13 individuals with confirmed rheumatoid arthritis (RA) to measure HCQ concentration levels. The study included individuals at least 18 years old who had been taking HCQ for at least six months at daily doses ranging from 200 to 400 mg. The Security Forces Hospital IRB committee approved the study (protocol/serial number: H-01-R-069, 20-434-46) and obtained informed consent from all study participants.

### 2.3 Calibration curve

HCQ calibration standards were prepared at six levels ranging from 20 to 2000 ng/mL (20, 60, 100, 500, 1500, 2000 ng/mL) for the assessment of the linearity of the method in LC/MS/MS. Chloroquine (CLQ) was used as an internal standard. Calibration standards and samples were freshly prepared on the day of the experiment. The linearity of the calibration curves was assessed by linear regression.

### 2.4 Sample preparation

To determine the mean plasma concentration of HCQ in patients, we collected plasma samples from 13 HCQ users using vacutainer tubes containing EDTA as an anticoagulant. Samples were kept at −80 °C until analysis. Protein precipitation extraction was used to treat plasma samples. Briefly, 50 µl of CLQ (100 ng/mL) was added to 500 µl of plasma samples or calibration standards. Then, 1.5 mL of acetonitrile (ACN) was added, followed by 30 s of shaking. The mixture was centrifuged at 10,000 rpm for 10 min, and 1 mL of the supernatant solution was transferred to an autosampler vial. For quantitative analysis, 7.5 µl of the prepared samples was injected into the LC/MS/MS.

COVID-19 incidence and complication data were gathered from participants and confirmed using data from the Saudi Ministry of Health's National Health Laboratory.

### 2.5 Chromatographic conditions

In this research, the concentration of HCQ in human plasma was determined using CLQ as an internal standard utilizing a validated LC/MS/MS (LC: Waters Acquity, Milford, MA, USA). Chromatographic conditions comprised the use of an Agilent SB-C8 column (50 mm × 2.1 mm, 3.5 m) with a mobile phase of 0.1% formic acid (FA) and ACN (30: 70 v/v) in an isocratic elution at a flow rate of 0.6 mL/min over a three run time. Tandem mass spectrometry using a TQ detector (Waters Corp., Milford, MA) equipped with a positive ionization electrospray ionization source (ESI) was used to identify the eluted components.

### 2.6 Statistical analysis

Data are reported as the mean ± SD for continuous variables. We used Student’s *t* test to compare the study groups using GraphPad Prism 9 software version 9.3.1.

## 3 Results

### 3.1 Method performance and assay validation

**Fig. 2:**
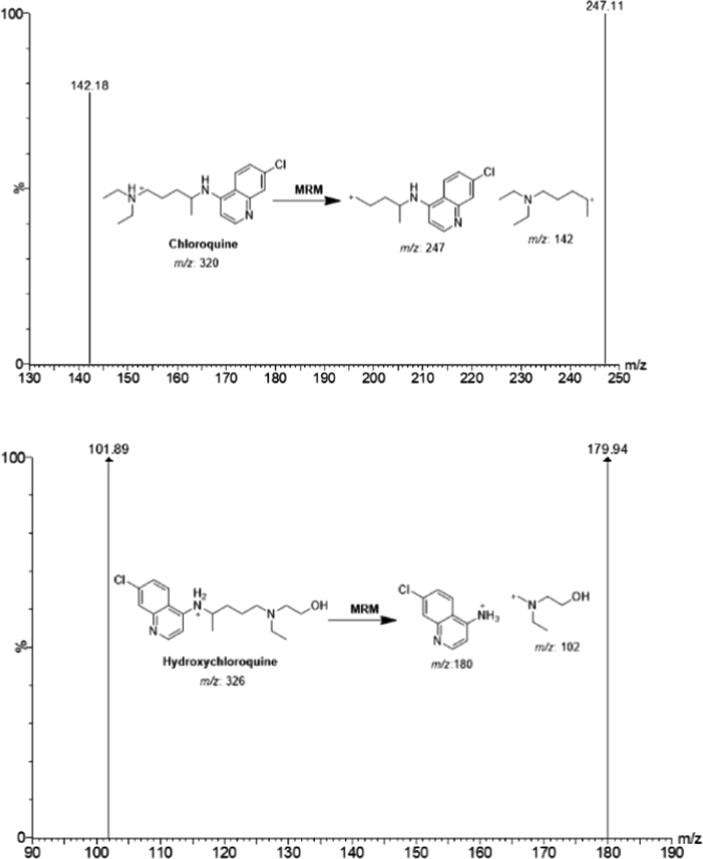
MRM mass spectrum of chloroquine and hydroxychloroquine showing its fragmentation pattern.

The quantification was carried out using multiple reaction monitoring (MRM) mode. The selection of ionization pairs (*m*/*z*) was presented as follows: CLQ: 320 → 247 and 320 → 142 (cone voltage 24 V, collision energy 20 V), HCQ: 326 → 180 and 326 → 102 (cone voltage 30 V, collision energy 34 V). MRM mass transitions are displayed in Fig. 2.

**Fig. 3:**
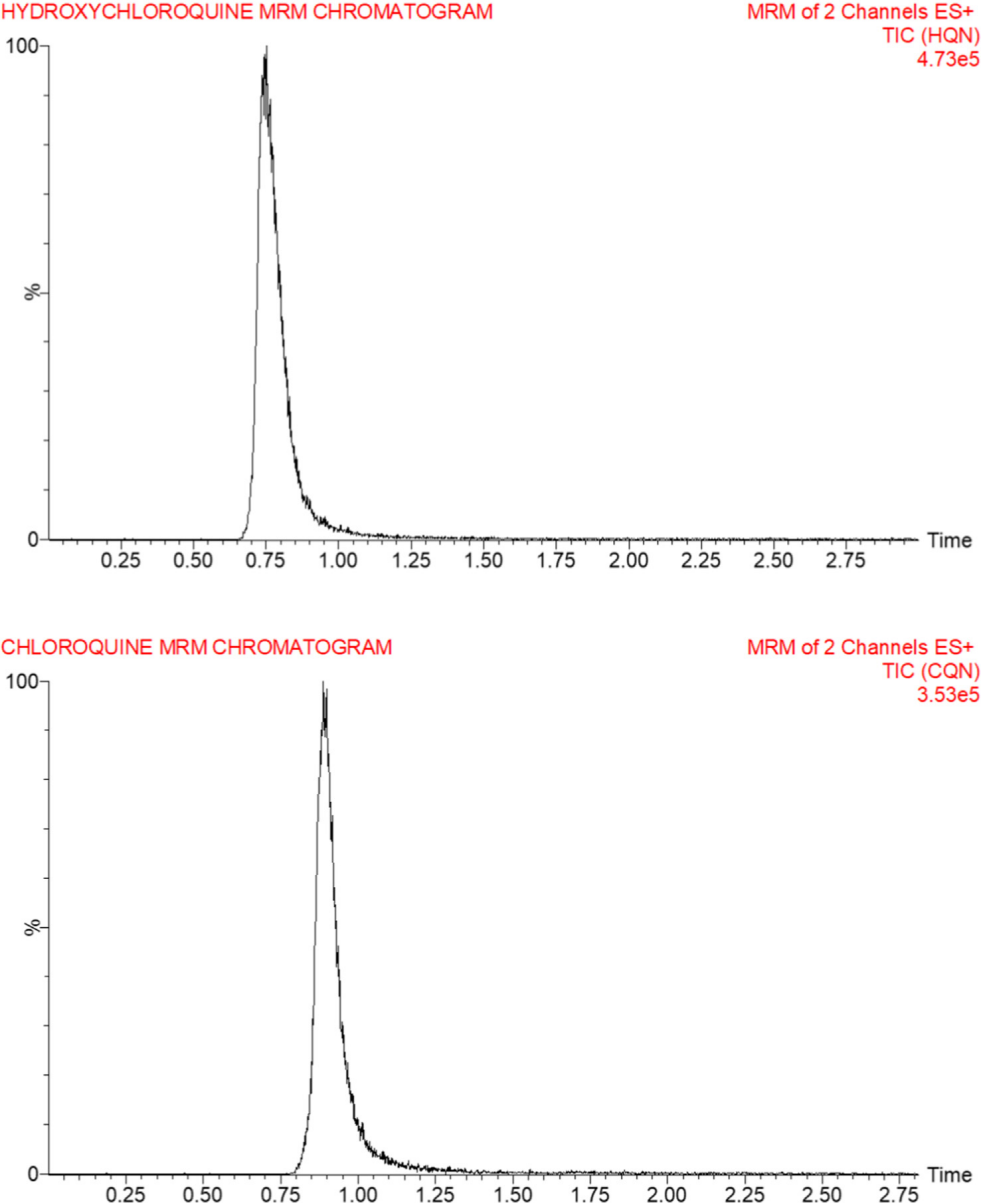
MRM chromatogram of hydroxychloroquine and chloroquine.

As potential mobile phases, we evaluated several combination ratios of ACN and 0.1% FA. CLQ and HCQ were efficiently separated in an isocratic elution program using the combination of ACN and 0.1% FA (70:30 v/v) (Fig. 3). Under the described chromatographic conditions, the retention time was approximately 0.75 and 0.89 for HCQ and CLQ, respectively. Both compounds were eluted without any endogenous interference from the blank human plasma.

### 3.2 Validation of the LC/MS/MS method

**Fig. 4:**
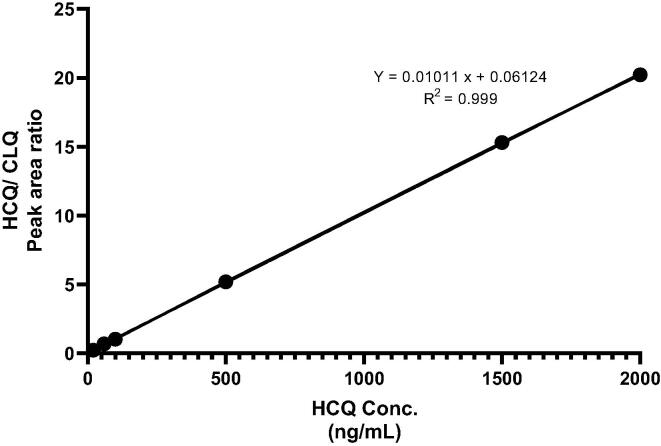
Calibration curve of hydroxychloroquine (HCQ). (Chloroquine (CLQ) concentration: 100 ng/mL).

We have developed and validated a method for estimating HCQ in human plasma using LC/MS/MS. The good linearity (r^2^ greater than 0.99), observable for HCQ over the range 0.02–2 µg/mL, has been described by the following regression equations: y = 0.0101x + 0.0604 (R^2^ = 0.9999), where Y refers to the peak area ratio of the drug to the internal standard, and X represents the analyte concentration in ng/mL in the plasma. The calibration peaks are illustrated in Fig. 4 and Table 2. The linearity of the analytical method was verified, as shown in Table 1. The lower limit of detection (LLOD) was calculated as (LLOD = 3.3σ / S), and the lower limit of quantification (LLOQ) was calculated as (LLOQ = 10σ / S), as σ is the standard deviation of the response and S is the slope of the calibration curve (REF). From the developed calibration curve, the LLOD was 6.4 ng/mL and the LLOQ was 19.3 ng/mL.

**Table 1:** Precision and accuracy of the developed method for hydroxychloroquine LC/MS-MS method.

Conc. (ng/mL)	Mean	SD	Precision	Accuracy	Recovery
20.00	16.85	0.28	1.69	−15.74	84.26
60.00	61.20	1.06	1.74	2.00	102.00
100.00	97.08	1.25	1.29	−2.92	97.08
500.00	504.14	3.38	0.67	0.83	100.83
1500.00	1531.40	17.73	1.16	2.09	102.09
2000.00	2017.16	25.80	1.28	0.86	100.86

**Table 2:** Calibration curve details of hydroxychloroquine (HCQ). (Chloroquine (CLQ) concentration: 100 ng/mL).

HCQ standard conc. ng/mL	HCQ area	CLQ area	Peak area ratio	Recovery
20.00	485.00	2135.00	0.23	16.51
60.00	1435.00	2125.00	0.68	60.88
100.00	2185.00	2132.00	1.02	95.49
500.00	11128.00	2145.00	5.19	507.67
1500.00	32102.00	2098.00	15.30	1508.99
2000.00	42917.00	2123.00	20.22	1995.53

### 3.3 HCQ plasma concentration

We collected plasma samples from 13 participants diagnosed with rheumatoid arthritis to study the effect of chronic HCQ use on COVID-19 infection (Table 3). Drug plasma concentration determines its pharmacological activity and can explain the different treatment outcomes in some cases.

**Table 3:** Demographic conditions of study participants.

	HCQ 200 mg daily dose		HCQ 400 mg daily dose	
	COVID-19	No COVID-19	COVID-19	No COVID-19
n =	3	4	3	3
Gender (M/F)	0/3	2/2	1/2	0/3
Age (years)	49.3 ± 7.8	39.5 ± 5.5	36.5 ± 4.5	34.0 ± 15.9
COVID-19 symptoms	Fever, Tiredness, Headache	N/A	Fever, Tiredness, Headache, loss of taste or smell	N/A

**Fig. 5:**
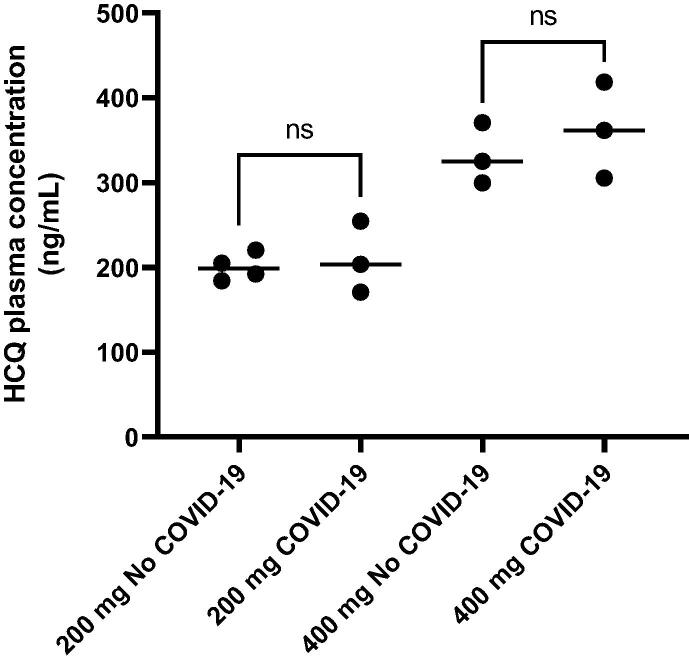
Hydroxychloroquine plasma concentration in patients diagnosed with COVID-19 or not. Doses were 200 mg – 400 mg daily. Differences among groups were analyzed using a student’s *t*-test. (ns: not significant, p-value < 0.05).

All patients were chronic users of HCQ (more than six months), ensuring that the drug reached a steady-state concentration, with limited fluctuation in drug concentration. All COVID-19 data were confirmed by the patients through the national health laboratory, Ministry of Health. The plasma concentrations of HCQ in all subjects are shown in Fig. 5.

The validated method described above was successfully used to determine HCQ concentrations in rheumatoid arthritis patients. This study enrolled a total of 13 patients (Table 3). All patients were chronic HCQ users, ranging in age from 21 to 69 years (median 45 years). The majority of patients (76%) were female, with a median body weight of 69 kg (range, 52–75 kg). Among the 13 patients, 7 patients were receiving HCQ at a dose of 200 mg per day, and 6 patients were receiving HCQ at a dose of 400 mg per day. COVID-19 confirmed cases accounted for approximately 46% of all patients. Half of the infected patients (n = 3) were taking a daily dose of 200 mg daily, while the other half were taking 400 mg daily. COVID-19 symptoms ranged from mild to moderate, and the intensity of the symptoms was not severe enough to necessitate hospitalization. COVID-19 symptoms in RA patients included headache, fever, fatigue, dry cough, and loss of taste or smell.

Blood samples were collected to ascertain whether insufficient HCQ concentrations are responsible for SARS-COV-2 infection. According to the measurement of plasma concentrations of HCQ at the time of blood collection (n = 13), the mean plasma concentration of RA patients taking 200 mg daily was 200.45 ± 15.73 ng/mL in patients without COVID-19, which did not differ from that seen in patients with COVID-19 (mean 209.66 ± 42.04 ng/mL). For RA patients receiving HCQ at a daily dose of 400 mg, the mean plasma levels of HCQ were 331.87 ± 35.97 ng/mL compared to those with COVID-19 (mean 361.79 ± 56.65 ng/mL) (Fig. 5).

## 4 Discussion

Our study examined a small population of patients with rheumatoid arthritis to determine whether HCQ concentrations in human plasma play a significant role in protecting RA patients against COVID-19 infection. When comparing patients with rheumatological conditions receiving hydroxychloroquine to those who did not receive hydroxychloroquine, the number of patients with confirmed COVID-19 cases was not different. We found no evidence that HCQ plasma concentrations were associated with either preventing COVID-19 incidence or reducing its complications. Additionally, we discovered that the COVID-19 symptom outcomes were similar in those receiving 200 mg and 400 mg daily. COVID-19 incidence or its complications are related to either low HCQ concentrations or the fact that HCQ has no impact on COVID-19.

Numerous randomized trials have demonstrated that HCQ has little therapeutic advantage when used to treat rather than preventing COVID-19 (Abella et al., 2021, Grau-Pujol et al., 2021, Rajasingham et al., 2021, Rentsch et al., 2021). As hydroxychloroquine has been shown to inhibit SARS-CoV-2 in vitro, it was investigated as a potential COVID-19 treatment. Several trials showed that HCQ and azithromycin significantly improved nasal clearance of SARS-CoV-2 compared to those patients without the combination of drugs (Gautret et al., 2020a, Gautret et al., 2020b, Rosenberg et al., 2020). The evidence of its effectiveness against SARS-COV-2 in *in vitro* and clinical studies led to widespread use of HCQ around the world. The results from previous studies are what led us to assume that there is a relationship between the concentration of hydroxychloroquine and the prevention of COVID-19 or its complications. However, our findings are consistent with a recent study, indicating that preexposure prophylaxis of HCQ for individuals whose median HCQ concentration reached 200 ng/mL did not significantly prevent SARS-CoV-2 infection among health care workers (Rajasingham et al., 2021). Another randomized clinical trial evaluated HCQ's efficacy against COVID-19 by administering a 600 mg daily dosage to participants for eight weeks. The results indicated that a higher dose of HCQ is ineffective in preventing COVID-19 (Abella et al., 2021). However, HCQ is less likely to cause side effects than CLQ. However, increasing the daily dose prior to exposure may result in adverse effects that are typically associated with long-term use. The severe adverse effects of HCQ on the system include retinal toxicity, neuromyotoxicity, and cardiotoxicity (Stokkermans et al., 2021).

The study's strength is that participants were regular HCQ users who were prescribed dosages consistently in clinical practice, with clear indications that these doses were administered at least six months prior to SARS-CoV-2 exposure. In most clinical trials, HCQ is administered at a daily dose of 200 mg or 400 mg, which is the same as the dose employed by our study participants. Our findings of HCQ plasma concentration levels were consistent with those published in previous studies (Mok et al., 2016, Balevic et al., 2019).

The study's primary weakness is its small sample size. Recruiting people was one of the challenges we encountered. We recruited participants (n = 13) over a six-month period. Due to the small sample size, it is difficult to determine the benefits and feasibility of implementation on a societal level. Another issue is the potential of residual confounding associated with the use of disease-modifying antirheumatic drugs (DMARDs).

## 5 Future recommendations

Since hydroxychloroquine has been repurposed as a treatment for viral diseases such as SARS, there must be a clear guideline for its reuse. Even if a vaccine for COVID-19 exists, we recommended that it be necessary to complete preclinical studies to clarify its effectiveness and whether it can be used in the future for viral diseases. Moreover, guidelines and databases that allow for the documentation of patient responses to HCQ used during the pandemic should be established.

## 6 Conclusion

The finding reveals that there is no evidence that HCQ plasma concentrations are correlated with reduced outcomes or the prevention of SARS-CoV-2 infection in rheumatoid arthritis patients with proven COVID-19. Despite the study's small sample size, our findings contribute to the growing body of evidence that HCQ is ineffective at preventing or mitigating COVID-19 infection. Currently, the SARS-CoV-2 vaccines provide hope; however, the emergence of variants highlights the importance of our study, which was to assess the effectiveness of HCQ in preventing diseases. We believe that further studies on large populations are required to evaluate the efficacy of HCQ in COVID-19.

## Funding

King Abdulaziz City for Science and Technology (KACST) funded this research through the fast-track funding pathway for Coronavirus (COVID-19) (grant number 5-20-01-016-0002).

## Institutional Review Board Statement

Ethical approval to conduct this study was received by Security Forces Hospital, Riyadh, Saudi Arabia, protocol/serial number: H-01-R-069, 20-434-46. This trial has received all ethical approval requirements from the appropriate ethical committees as described above.

## Informed Consent Statement

Written informed consent was obtained from subjects involved in this study.

## Declaration of Competing Interest

The authors declare that they have no known competing financial interests or personal relationships that could have appeared to influence the work reported in this paper.
